# Translating technological innovation into efficiency: the case of US public P&C insurance companies

**DOI:** 10.1007/s40821-021-00189-7

**Published:** 2021-09-13

**Authors:** Davide Lanfranchi, Laura Grassi

**Affiliations:** School of Management, Politecnico di Milano, Milan, Italy

**Keywords:** Efficiency, Insurance, Technology, Insurtech, Competition, Innovation

## Abstract

In recent years, Insurtech innovations, driven by technologies such as artificial intelligence and blockchain, emerged in the insurance industry, with the promise of improving efficiency. However, while the positive impact of technology on insurance companies’ efficiency is expected, literature assessing it empirically is scarce, when it comes to recent technological change. Focusing on the US public P&C insurance sector in the period 2012–2018 and relying on both nonparametric (two stage DEA) and parametric (SFA) approaches, it emerges that on average insurance companies were not able to leverage on technological innovations to improve their efficiency. On average a relative level of efficiency among companies, according to a two stage DEA model, was quite stable in time, while the SFA approach shows that the distance between efficient and less efficient firms slightly increased. Moreover, we found one very efficient firm, almost a leader of the market in terms of efficiency, and a homogeneous group of followers, indicating that there is vast scope for improvement for less efficient companies. Nevertheless, even the most efficient company impaired its efficiency over time, suggesting that neither the leader nor on average the followers properly leveraged technology to improve their efficiency. In a competitive scenario, with new players’ entrance and fierce competition, inertia may seriously affect their positioning. Academicians, managers and policymakers should carefully consider the effects that a non-improvement of efficiency following technological change may have on market structure, competition and regulations, potentially opening to further discussion on how technological innovations adoption should be facilitated.

## Introduction

“In a world of increasing uncertainty and dynamics, the economic and social importance of being insured seems undisputed” (Stoeckli et al., 2018, p. 287). The recent pandemic related to COVID-19 revealed to the public the relevance of the insurance industry for its impact on daily life. From an economic point of view, insurance is fundamental by dealing with the negative consequences of economic activities that would occur in its absence (Zweifel & Eisen, 2012), while from a social point of view, insurance is crucial by providing social protection mechanisms (The Geneva Association, 2012), in turn positively affecting country’s economy (Grmanová & Strunz, 2017). Due to their conservative attributes, insurance companies traditionally struggled with innovation (Nam, 2018). However, new developments and changes in society and in economy affect the demand for insurance (Bohnert et al., 2019), and considering socio-economic changes to which it is connected in several ways (Bohnert et al., 2019), the insurance industry needs to innovate. In recent years, we are observing the increasing relevance and disruptive nature of digitalization (Fitzgerald et al., 2014) and the emergence of Insurtech, “a phenomenon comprising innovations of one or more traditional or non-traditional market players exploiting information technology to deliver solutions specific to the insurance industry” (Stoeckli et al., 2018, p. 289). Interest in the possibilities arising from digital innovation (Deloitte, 2018), focusing in particular on the impact that new technologies such as artificial intelligence (McKinsey, 2018a), blockchain (BCG, 2018) and big data (Corlosquet-Habart & Janssen, 2018) may have on the increase of efficiency (McKinsey, 2018b) is noticeable for traditional insurance companies, practitioners and academicians.

Academic literature highlights how recent technological innovation in the insurance sector brings with it the promise of increasing efficiency (Lin & Chen, 2020): for instance, artificial intelligence can bring efficiency in the personalization of insurance policies (Al-witwit & Ibrahim, 2020); blockchain, by disintermediating, could bring in a more efficient approach by increasing speed and reducing costs (Grima et al., 2020); online interfaces and virtual claims adjusters could make the settling and payment of claims following an accident more efficient, concurrently decreasing the likelihood of frauds (Clemente & Marano, 2020). However, despite the contributions suggesting that a positive effect of new technologies emerged in the last decade on insurance companies’ efficiency, literature trying to further investigate this relation is scarce. Not just academicians, but also managers and policymakers should carefully consider the effects that a (non)improvement in efficiency following technological change may have on the market structure, competition and regulations, potentially opening to further discussion on how technological innovations should be grounded and effectively adopted or facilitated.

For this reason, this paper examines with an empirical approach whether insurance companies improved their efficiency, by relying on new technologies.

To do so, the scope of this work is the public property and casualty (P&C) insurance sector in the United States (US). The US insurance market is the largest in the world in terms of total direct premium volume, with a global market share of 39% in 2019 (Swiss Re Institute, 2020). Furthermore, the United States is the most advanced country in terms of technological innovation in the insurance sector, as suggested for instance by the relevance of the Insurtech startups ecosystem in this country; in terms of investments in Insurtech startups, the US leads the ranking, with nearly 45% of world Insurtech startups able to attract nearly 50% of total investments (NTT Data, 2020). Of the two main kinds of insurance, Life insurance, where the insurer invests the insured’s savings undertaking to pay a capital or an income in case of a life event, such as death; and P&C—Property and Casualty insurance, that protects the insured against possible losses deriving from damages to persons or objects (Marchionni, 2006), we concentrate on insurance companies solely in the P&C insurance business. The P&C segment is particularly detrimental for the market as it deals with almost any kind of risk (Ilyas & Rajasekaran, 2019) affecting daily and business continuity. Indeed, process digitalization (such as for sales and distribution) and achievement of higher efficiency are among the top trends in the P&C market according to practitioners (EY, 2019), with the promise of promoting efficiency in processes that are particularly relevant for this kind of insurance (e.g. claim settlements, Clemente & Marano, 2020). In this context, the US P&C insurance sector in particular had a relevant growth over the past years: in 2018, this sector has seen a net income increase of 66% to US$60 billion, thanks to a 10.8% boost in net premiums (Deloitte, 2019).

Therefore, focusing on the US P&C public insurance sector, the rest of the paper reviews extant literature presenting theoretical and empirical findings on the effect of technological improvement on efficiency. Attention to methodological issues and description of the sample anticipate the discussion of results and conclusions.

## Literature review

Examining the relation between technological change and efficiency requires some elements of theory, hereby presented to introduce empirical evidence and approaches to estimate the relation.

### Efficiency and technological change: theoretical background

Efficiency is a condition for survival in a competitive scenario (Fried et al., 1993; Mogos et al., 2021), opening the way for a firm’s outperformance in the market (Schaeck & Cihák, 2014), feeding its stability (Schaeck & Cihák, 2014). Technology contributes to productivity (Bartelsman et al., 2019), efficiency (Voghouei & Jamali, 2018) and thus to the ability of the companies to compete (Battese & Rao, 2002; Sonenshine, 2020).

However, even well managed companies may lose their dominance in the market, failing when disruptive changes in technology emerge (Christensen, 2013). Such a failure can be related to the companies’ inability to consider new disruptive technologies in a timely manner, but also to their inability to commercialize them successfully (Vecchiato, 2017). When it comes to insurance companies, the industry is experiencing a clear change (McKinsey, 2018b) for the increasing relevance of digitalization (Fitzgerald et al., 2014) and the emergence of Insurtech. Recent technological progress deals not only with the technology itself, but also with the availability of information, affecting the playing field in informationally sensitive markets (Hauswald & Marquez, 2003). Technological innovation in the insurance sector brings several benefits such as better understanding of underlying insurance risks and increasing efficiency and lowering costs for insurers, intermediaries, and customers (Lin & Chen, 2020), contributing, as innovation in general, to economic growth (Pellegrino & Piva, 2020).

### Efficiency and technological change: empirical evidence

Efficiency measurement is a fast-growing area in business and economics literature (Biener et al., 2016) and different recent contributions analyzed efficiency of P&C insurance industry, adopting from time to time different geographical perspectives, from national (e.g. South Africa, Alhassan & Biekpe, 2016) or India (Ilyas & Rajasekaran, 2019)) to multi-country (e.g. Europe, as in Jarraya and Bouri, 2015). Some studies analyzed the efficiency of the US insurance industry (e.g. Copeland and Cabanda, 2018), focusing on 2011–2013, and Cummins and Xie, 2016, focusing on 1993–2011). Eling and Luhnen (2010) and Ferro and Leòn (2018) provided a review on studies analyzing efficiency in the insurance sector. In several cases, results show that there is significant room for improvement in terms of efficiency of insurance companies, as their actual level is moderate to low (Cummins & Xie, 2016; Ilyas & Rajasekaran, 2019; Worthington & Hurley, 2002), even compared to other financial segments (Cummins, 1999). Said otherwise, there is substantial room for improvement in efficiency. A further stimulus for increasing efficiency came from relevant technological innovations emerging in the insurance panorama (Lin & Chen, 2020), in particular starting from 2012 (Willis Tower Watson, 2018), that were capable of making relevant insurance processes more efficient (Clemente & Marano, 2020). Some contributions had the purpose of studying the evolution of efficiency during time due to technological change. Ferro and León's (2018) results show that the productivity of the industry was not improving over the years, and one of the causes was the non-technological improvement. Companies were not investing in technology or the investments turned in no positive effect on productivity (premiums) (Ferro & León, 2018).

Despite the presence of works studying efficiency levels in the insurance industry in recent years (Camino-Mogro & Bermúdez-Barrezueta, 2019; Nguyen & Worthington, 2020), they do not further investigate the effect of technological change. In the light of previous results, showing a moderate efficiency in the industry and of recent technological innovations with the potential to change the industry (Eling & Lehmann, 2018), in particular in the country mostly affected by innovative technological applications in insurance [i.e. the US (NTT Data, 2020)], we deem very relevant to contribute to the understanding of the effect that recent technological innovations have, potentially challenging the validity of former results and affecting the dynamics of market players’ competition. To the best of our knowledge, there are no timely contributions on insurance companies’ efficiency and on the effect of technological change focused on the US in recent years (post-2012).

### Efficiency and technological change: measures, methods and approaches

Measurements support us in quantifying such theoretical underpinnings (Fried et al., 1993). To estimate efficiency, the emergence of frontier methodologies has been a relevant development in modern economics, encompassing the limitations of financial ratios (Huang & Eling, 2013). The basic idea is to identify efficient^1^ companies, namely companies that maximize their output considering the inputs at their disposal, with respect to non-efficient ones. The group of efficient companies forms the efficient frontier. Hence, for all companies, efficiency is measured with reference to a frontier consisting of the dominant companies in the industry. Frontier efficiency measures summarize a company’s efficiency in a single measure (values from 0, non-efficient company, to 1, fully efficient company) that checks for differences among companies according to a sophisticated multidimensional framework that has its roots in economic theory (Cummins & Weiss, 2013) and implies some reasoning on the production function. However, new or innovative technological processes appear over time, these imply different ways of combining inputs, or different ways of combining processes (technology set). Any enlargement of the corresponding technology set is, by definition, a technological change (Gomulka, 2006). The addition of an efficient technology moves the production frontier further outwards, and thus any such movement indicates the presence of technological progress within the company (Gomulka, 2006).

The existing different approaches focus on the estimation of the production function, following either a parametric or non-parametric approach. Danquah et al. (2018) and Delhausse et al. (1995) applied parametric techniques, stochastic frontier analysis (SFA) in particular, which provide techniques for modeling the frontier within a regression framework in order to estimate efficiency. Other authors applied non-parametric techniques, which utilize linear programing techniques to estimate the frontier and provide relative assessment (Tuzcu & Ertugay, 2020) such as Data Envelopment Analysis (DEA) (Barros et al., 2005; Cummins & Turchetti, 1996; Hesarzadeh, 2020; Nguyen & Worthington, 2020; Nourani et al., 2020; Shieh et al., 2020), and two-stage DEA (Li et al., 2018). The choice of the methodology for estimating efficient frontiers has generated debates in the literature, with some scholars supporting the parametric approach (Berger, 1993; Greene, 2008) and others the nonparametric one (Cooper et al., 2011), with no clear conclusion. The main advantage traditionally offered by the parametric approaches (hence SFA) in comparison with the nonparametric ones (such as DEA) is that SFA allows to distinguish randomness from efficiency (Ferro and Leòn, 2018). On the other hand, the primary advantage traditionally given by nonparametric approaches as DEA is to avoid misspecification of the functional form or the probability distributions assumed for error terms, which could confound the efficiency estimates with specification errors. Some authors are then opting for using both techniques (e.g. Ur-Rehman et al., 2020), to leverage on advantages of both approaches.

## Methodology

This work studies efficiency in the US P&C public insurance sector by estimating the production function, which connects the level of output, given the inputs, focusing on technical efficient companies, namely the ones capable of producing the highest level of output conditional on input use levels, consistently with previous literature (Ferro and Leòn, 2018). Therefore, observed output $(y_{i} )$ is connected to the production function $f\left( {{\varvec{x}}_{i} ;\beta } \right)$ and to input $\left( {{\varvec{x}}_{i} } \right)$ as:

$$y_{i} = a_{i} f\left( {{\varvec{x}}_{i} ;\beta } \right),\quad 0 < a_{i} \le 1$$

In literature, different approaches for identifying outputs and inputs co-exist.

### Output measure

As reported by Cummins and Weiss (2013), there are three main approaches to measure outputs in financial services—the asset intermediation approach, the user–cost approach, and the value-added approach. The intermediation approach considers financial companies as pure financial intermediaries and consider assets as outputs. This approach is inappropriate for P&C insurers as they provide further services in addition to financial intermediation. The user–cost method determines whether a financial product is an input or output depending on its net contribution to the revenues. This approach is problematic for the insurance industry owing to policies comprising many services, which are priced implicitly. Under the third approach—the value-added approach— categories having significant value-added are employed as important outputs: this approach is widely adopted in literature (Delhausse et al., 1995; Fecher et al., 1993; Fuentes et al., 2005; Rai, 1996), and current research relies on this approach as well, with premiums as output.

### Input measure

Concerning input variables, consistently with Rai (1996), variables adopted in this work are claims, labor, and capital. The evaluation of the premiums that will be collected from insured entities (customers) (y_i_) starts from estimating the so-called fair premium, hence the amount needed to cover the expected losses that the customer may suffer during the protection period and that the insurance company will have to repay (hence, the claims) (Zweifel & Eisen, 2012). Claims are hence considered as inputs, being a determinant of the amount of premiums that an insurance company will collect at the beginning of the protection period. Along with the claim’s current value, we considered the reserves, which refer to the estimated subsequent compensations costs, the related direct and settlement costs, the provision for late reporting and, more in general, the charges deriving from the part of the risk not yet concretized. On top of it, premiums loadings are charged to count for other expenses, such as operating and administrative expenses, which we will consider under the labor input. As insurance companies sell their policies through agents relying on their own staff for back-office work (Ferro and Léon, 2018), labor is composed mainly by brokers’ labor (accounting for the larger part of commissions) and home office labor (hence wages). Finally, to perform insurance activities, regulators require a minimum of equity capital from insurance companies (Zweifel & Eisen, 2012), to insure solvency also in case of unexpected losses. Capital, the third input considered, has its roots in Adam Smith’s Wealth of Nations as “In order to give this security, however, it is necessary that the insurers should have a very large capital” (Smith, 2007, p. 586).

### Production function estimation

To estimate the production function and to cross-check our results, we adopted both a nonparametric (two stage—DEA) and a parametric approach (SFA), in the light of the above-mentioned benefits and drawbacks of each technique.

#### Two stage—DEA

DEA is a method that allows measuring efficiency of companies relying on linear programing techniques, to envelop observed input–output vectors as tightly as possible to build the efficiency frontier (Boussofiane et al., 1991). By adding weight constraints, DEA models can be subdivided in terms of returns to scale. Charnes et al. (1978) originally proposed the efficiency measurement of companies for constant returns to scale (CRS) assuming all companies were operating at their optimal scale. Banker et al. (1984) introduced the variable returns to scale (VRS) efficiency measurement model, allowing hence the breakdown of efficiency in DEA into technical and scale efficiencies. The concept of frontier is important for the analysis of efficiency, as we measure efficiency as the relative distance from the frontier. DEA models can be divided into input-oriented models, which minimize inputs while respecting the given output levels, and output-oriented models, which maximize outputs without requiring higher input quantities. In both cases, efficiency is measured in terms of a proportional change in inputs or outputs.

Companies that are technically not efficient operate at points in the interior of the frontier and will have a DEA score lower than 1. In the input-oriented model, the score indicates the percentage of input that the company should use to become efficient, given a certain output level. In the output-oriented model, the score is the output produced by the non-efficient company in percentage of the output produced by an efficient one. A company is called “radial” or “weak” efficient when the DEA score is equal to 1. If, along with this, all slacks [a slack issue arises as the frontier runs parallel to the input or output axes resulting in input/output excesses (Lee & Ji, 2009)] are zero, the company is called efficient in terms of “Pareto–Koopmans” or “strong” efficiency. When the slack issue is neglected and calculated residually, the DEA model becomes the single-stage DEA model; to the contrary, two-stage DEA directly faces slacks issues.

To evaluate technological change under the DEA nonparametric approach, it is useful to rely on Malmquist Productivity Index (MPI) that measures the productivity changes in time and can be decomposed to isolate the technology-driven change (Lee et al., 2011). In particular, a technological change factor higher than 1 means that the company was able to improve its technology to gain efficiency, while a factor lower than 1 means that the technological set deteriorated in terms of effects on efficiency.

#### SFA

The basic empirical framework for SFA is a regression specification involving a logarithmic transformation of the production function that adds a random error term (v_i_), where output is bounded from above by the stochastic frontier $f\left( {{\varvec{x}}_{i} ; \beta } \right)e^{{v_{i} }}$, and $u_{i} = - \ln \left( {a_{i} } \right) \ge 0$ represents unit specific technical inefficiency. Hence, technical efficiency is recovered as $e^{{u_{i} }}$

$$ln y_{i} = ln f\left( {{\varvec{x}}_{i} ;\beta } \right) + v_{i} - u_{i} .$$

The application of a SFA technique requires the discussion and choice of a functional form of production function, $f\left( {{\varvec{x}}_{i} ;\beta } \right)$, and of a model for unit specific inefficiency, $u_{i}$.

For the functional form of production function, this work will test two well-known forms in literature, the Cobb–Douglas function in the logarithmic form, because of its simplicity and its easy interpretation (Ferro and Leòn, 2018), and the trans-logarithmic function, a more flexible functional form than Cobb–Douglas (Cummins & Weiss, 2013).

As mentioned, an efficient technology moves the production frontier further outwards (Gomulka, 2006): this change is captured by a linear indication of time in the Cobb–Douglas function and by a quadratic polynomial of time in the trans-logarithmic function. The rate of technological change is given by T* = δy/δt, considering time affecting efficiency due to technological change. If T* > 0, technological change is positive, indicating a growth in efficiency, and vice versa.

For unit specific inefficiency functional forms, we will test a version of stochastic frontiers with time-invariant inefficiency and one with time-varying inefficiency. Regarding time-invariant inefficiency, Battese and Coelli (1988) (BC88) proposed a maximum likelihood (ML) estimation of the following Normal-Truncated Normal model:

$$y_{it} = \alpha + x_{it}^{\prime } \beta + \varepsilon_{it} ,\quad i = 1, \ldots ,N,\quad t = 2, \ldots ,T_{i}$$

$$\varepsilon_{it} = v_{it} - u_{i}$$

$$v_{it } \sim N\left( {0,\sigma_{v}^{2} } \right)$$

$$u_{i } \sim N^{tr} \left( {0,\sigma_{u}^{2} } \right)$$

Regarding time-varying inefficiency, Battese and Coelli (1992) (BC92) proposed a “time decay” ML estimated model for the unit specific inefficiencies:

$$u_{it} = g\left( t \right)u_{i} ,\quad g\left( t \right) = e^{{\left[ { - \eta \left( {t - T_{i} } \right)} \right]}}$$

where *T*_*i*_ is the last period in the panel for the unit *I* and the $u_{i }$ are assumed to be independent and identically distributed Truncated Normal. For η going to zero, this model converges to BC88. A negative η suggests that the relative levels of efficiency among companies is decreasing, hence the gap with the most efficient one is widening.

**Fig. 1:**
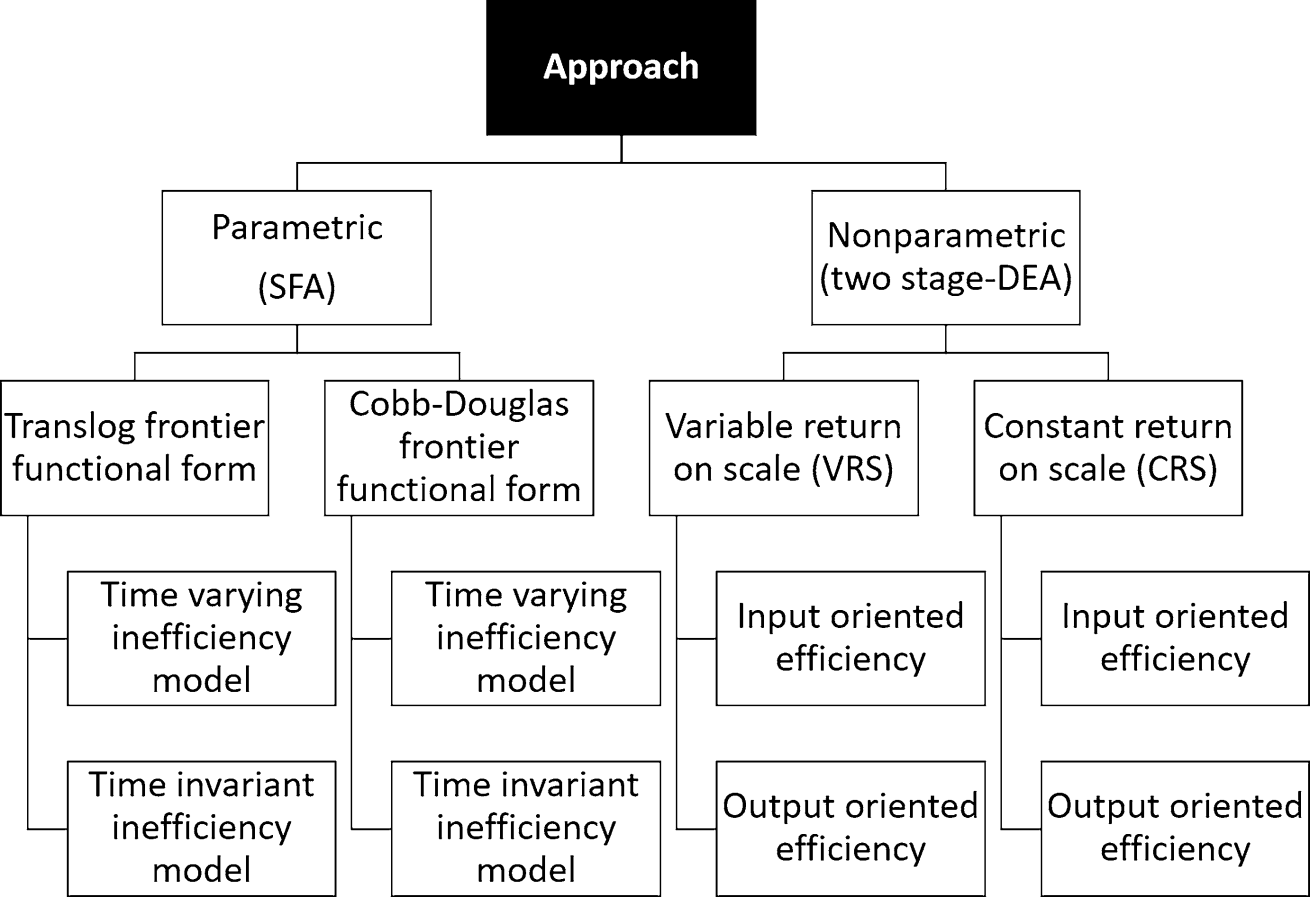
Summary of adopted approaches and models

To give strength to the results with respect to the specific choice of models, this work will hence rely on two different approaches, a parametric (SFA) and a nonparametric approach (two-stage DEA). As a result, for two-stage DEA four models will be involved (input and output oriented, both assuming CRS and VRS), while for SFA, four models will be tested (two functional forms for each production function combined with two models for unit specific inefficiencies, to address the mentioned criticality of parametric approaches of choosing functional forms for frontier and error distribution), as summarized in Fig. 1.

## Data collection and analysis

The measurement of input and output variables (see Table 1) reflects the structure of the sector and the way it is organized. Claim input includes both claims that are already paid as well as new reserves. Labor input includes both selling and general and administrative expenses, reflecting how insurance companies sell policies through agents, earning commissions, and relying on their own staff for back-office work (Ferro and Leòn, 2018). Finally, capital consists in total shareholders’ equity. Concerning premiums as a measure of output, we considered sales as a proxy. Data have been obtained from the FactSet Fundamentals database^2^ over the period 2012–2018. Indeed, starting from 2012, technological innovations emerging in the insurance panorama, such as blockchain, artificial intelligence and others (see Eling and Lehmann (2018) for a detailed list of such innovations) started receiving increasing attention (Willis Tower Watson, 2018). At the same time, investments in Insurtech startups raised from less than 500 USD million in 2012 to more than 2.5 USD billion in 2017 (Milken Institute, 2018), and more than 4 USD billion in 2018 (Willis Tower Watson, 2019).

**Table 1:** Summary of input and output variables

Variable	Type	Proxy (FactSet items)
Claims	Input	Losses, claims and reserves
Labour	Input	Selling, general and admin. Expenses and other
Capital	Input	Shareholders’ equity
Premiums	Output	Sales

The population of public US P&C insurance consists of 40 companies, as in FactSet Fundamentals. Given the non-availability of data in the analyzed period for 5 units, the final sample is a panel of 35 companies, with a representativeness of 99.84% in terms of collected premiums in 2018 of the overall population of public insurance companies active solely in the P&C business. The insurance companies in the sample are diversified in terms of dimensions (see Table 2): indeed, looking at premiums, average amounts of collected premiums are around 4.6 billion USD per year, ranging from a maximum of about 39 billion USD to a minimum of about 7 million USD.

**Table 2:** Descriptive statistics of input and output variables

	Premiums (M$)	Claims (M$)	Labor (M$)	Capital (M$)
Mean	4602	2848	1323	3728
Standard deviation	8181	5186	2274	6418
Max	39,124	25,466	11,196	25,405
Min	7	0	4	21
Observations	243	236	241	242

Moreover, considering the parametric technique (SFA), we controlled for Gross Domestic Product (GDP) (Outreville, 1990), interest rates (Beenstock et al., 1988), inflation (Boubaker & Sghaier, 2014), and concentration (Weiss & Choi, 2008), relying on the Gini index, in terms of 2018 collected premiums. In particular, as shown in Table 3, concentration in the market remained quite stable over time at around 70%.

**Table 3:** Control variables values over time

	GDP US (M$)^a^	1Y Interest rate^b^ (%)	Inflation^a^ (%)	Gini index^c^
2012	16,197,007	0.2	2.1	0.722
2013	16,784,849	0.1	1.5	0.730
2014	17,521,746	0.1	1.6	0.717
2015	18,219,297	0.3	0.1	0.720
2016	18,707,188	0.6	1.3	0.713
2017	19,485,393	1.2	2.1	0.719
2018	20,529,049	2.3	2.4	0.721

^a^World Bank

^b^US Dept. of the Treasury 1Y Treasury Bill US—avg Closing Price

^c^Authors’ calculations based on 2018 premiums

Table 4 shows correlations among the variables involved. As we can see, positive correlations are present between the output variable (premiums) and all the input variables (0.99 with claims, 0.96 with labor and 0.87 with capital). Control variables, on the other hand, are not particularly correlated with output and input variables.

**Table 4:** Correlations among input, output and control variables

	Premiums	Claims	Labor	Capital	GDP	Interest rates	Inflation	Gini index
Premiums	1.00							
Claims	0.99***	1.00						
Labor	0.96***	0.91***	1.00					
Capital	0.87***	0.80***	0.93***	1.00				
GDP	0.04	0.05	0.07	0.00	1.00			
Interest rate	0.04	0.06	0.07	0.00	0.89***	1.00		
Inflation	0.02	0.03	0.01	0.01	0.21***	0.53***	1.00	
Gini Index	− 0.01	− 0.02	0.00	− 0.00	− 0.39***	− 0.13**	0.10	1.00

***p value < 1%; **p value < 5%; *p value < 10%

## Results and discussion

This section presents and discusses main evidence resulting from nonparametric and parametric approaches, relying on the above presented data.

### Results with a nonparametric approach (two stage-DEA)

By applying a nonparametric technique, namely two stage-DEA, to analyze the effect of technological change on companies’ efficiency, it emerges how, during the considered period, on average the companies in the industry were not gaining efficiency by leveraging technology. Considering the technological change factor resulting from the Malmquist index (Table 5), it emerges how, despite an average improvement in technology from 2012 to 2013 (with a factor of 1.23), during the rest of the period the average technological quality in the industry remained the same in terms of effects on efficiency, and even slightly worsened during 2013–2014 and 2015–2016 (with factors respectively of 0.96 and 0.89). Hence, from 2013, on average the industry was not able to rely on technological innovations to improve its efficiency.

**Table 5:** Technological change factor over the analyzed period

	2012–2013	2013–2014	2014–2015	2015–2016	2016–2017	2017–2018
Mean	1.23	0.96	1.00	0.89	1.00	1.00
SD	0.28	0.09	0.03	0.08	0.02	0.06
Maximum	2.50	1.07	1.08	1.07	1.06	1.28
Minimum	0.97	0.53	0.96	0.77	0.94	0.89

Furthermore, it emerges that no company was able to improve its efficiency over the whole period (even if one company always improved except for once) nevertheless no company suffered a decrease of efficiency in each period (even if six of them were able to improve only once). Hence it is relevant to investigate how the relative level of efficiency among companies (that is, the efficiency of a company with respect to the most efficient ones) changed over time, to understand whether some companies performed better than the others in improving their efficiency, therefore widening the gap with the less efficient ones. From the four two stage-DEA models (input vs output oriented, combined with CRS vs VRS assumption) it emerges how on average the relative level of efficiency among companies did not change, remaining for all models well above 83% for the whole period, with less efficient companies never lowering their relative efficiency score under 58%, regardless the model (Table 6). We can therefore conclude that, according to two stage-DEA models, the sector was on average homogeneous in terms of efficiency and maintained this uniformity all along 2012–2018.

**Table 6:** Descriptive statistics of efficiency scores estimated with the two-stage DEA approach

	2012 (%)	2013 (%)	2014 (%)	2015 (%)	2016 (%)	2017 (%)	2018 (%)
CRS input oriented							
Mean	91	83	84	84	90	88	89
SD	7	12	11	11	8	9	8
Maximum	100	100	100	100	100	100	100
Minimum	79	58	62	58	72	62	66
VRS input oriented							
Mean	97	94	94	94	95	93	94
SD	5	9	8	8	6	7	7
Maximum	100	100	100	100	100	100	100
Minimum	84	70	75	71	84	80	76
CRS output oriented							
Mean	91	83	84	84	90	88	89
SD	7	12	11	11	8	9	8
Maximum	100	100	100	100	100	100	100
Minimum	79	58	62	58	72	62	66
VRS output oriented							
Mean	97	94	94	94	95	93	94
SD	4	8	8	8	6	7	7
Maximum	100	100	100	100	100	100	100
Minimum	85	70	75	73	84	78	76

### Results with a parametric approach (SFA)

When it comes to the parametric approach, to identify the most suitable model, we started comparing the two functional forms for the production function, namely the Cobb–Douglas and trans-logarithmic functions. The trans-logarithmic version was rejected (i) being most of the quadratic and interaction variables not significant, and (ii) considering Bayesian information criterion (BIC) and Akaike’s information criterion (AIC) results. Hence, focusing on the Cobb–Douglas functional form for the production function (time invariant and time varying models), Table 7 shows the relation between input and output variables. From both models the relation between claims and premiums collected by the insurance companies is positive and significant (p value < 1%): as mentioned, the estimation of the premium starts from estimating the so-called fair premium, hence the amount needed to cover only expected losses (Zweifel & Eisen, 2012). In the same way, the relation between labor and premiums is positive and significant for all models (p value < 1% for both models): as highlighted, labor force plays a major role in collecting premiums. Concerning capital, once more the relation with premiums is positive and significant for all models (p value < 1% for both models): capital, as mentioned, is fundamental to enabling insurance activity. It emerges from both models that the technological change had a slightly negative effect on the average industry efficiency: indeed, T parameter is equal to -0.0557 in BC88 (p value < 5%) and − 0.0174 in BC92. Hence, on average, the companies in the industry were not gaining efficiency by leveraging technology.

**Table 7:** Results of the estimations of SFA models (time invariant and time varying)

	Time invariant model (BC88)	Time varying model (BC92)
Frontier		
Ln (claims)	0.5340*** [0.0268]	0.5212*** [0.0317]
Ln (labor)	0.1679*** [0.0304]	0.1744*** [0.0300]
Ln (capital)	0.2379*** [0.0412]	0.2487*** [0.0429]
T	− 0.0557** [0.0280]	− 0.0174 [0.0368]
Ln (GDP)	1.6210** [0.7515]	1.5614** [0.7825]
Interest Rate	− 1.9399 [1.8231]	− 1.4837 [2.0633]
Inflation	− 1.1457** [0.5235]	− 1.1194** [0.5141]
Gini Index	1.7813* [0.9799]	1.7937* [0.9991]
Constant	86.0787* [44.4822]	9.8722 [62.2649]
η		− 0.0343* [0.0207]

***p value < 1%; **p value < 5%; *p value < 10%. Standard errors in []

The slightly negative (and significant, p value < 10%) value of η under BC92 (Table 7) suggests the time varying model to be the most appropriate and will hereby be considered for the discussion. As in nonparametric approaches, we investigated further the efficiency scores, to understand how the relative level of efficiency among companies has changed over time. It emerges that average efficiency levels are quite low (mean 35.2%, standard deviation 10.3%, Table 8), constantly decreasing in time, going from 37.8% to 32.1%, showing a slightly increasing efficiency gap between efficient and less efficient companies over time.

**Table 8:** Efficiency scores estimated considering a time varying model (BC92): average scores over time and descriptive statistics

	Average efficiency scores (%)	Descriptive statistics
2012	37.8	Mean: 35.2% SD: 10.3% Maximum: 93.3% Minimum: 16.8%
2013	36.3	
2014	36.8	
2015	35.5	
2016	34.3	
2017	33.3	
2018	32.1	

The range of efficiency scores among companies is very wide, going from very efficient companies (score higher than 93%, Table 8) to not very efficient ones (score of about 16%).

Considering the level of heterogeneity, a further investigation of the performance of each single firm shows a very efficient company, better than all the others: indeed, it emerges that, while the efficiency score of the most efficient unit is always well above 90%, considering the second most efficient unit, its score is never higher than 49%, about half of the efficiency compared to the most efficient one. These results suggest that we are dealing with an industry composed by one very efficient company, a kind of leader of the market in terms of efficiency, and a homogeneous group of followers.^3^ Therefore, less efficient companies have considerable scope for increasing efficiency, by improving the level of the output, given the amount of inputs, to close the gap with the most efficient company in the group. However, this highly efficient company decreased its efficiency over time (indeed, as suggested by the negative value of parameter T, technological change had the effect of decreasing the efficiency level of the frontier, of which this company is the leader). This, combined with the evidence of a slightly increasing gap, suggest that on average followers decreased their efficiency even further over time. In a competitive scenario, where new players are entering (namely Insurtech players) and competitors have to leverage technology to improve their efficiency, a situation of inertia may seriously affect the positioning of companies, both for the leader and for the followers.

### Comparing results from nonparametric and parametric approaches

To summarize, both the nonparametric approach (two-stage DEA) and the parametric approach (SFA) suggest that US public P&C insurance companies on average, in the period 2012–2018, were not able to leverage technological innovations to improve their efficiency. With a DEA approach, the average technology change index obtained from the Malmquist index was always lower than or equal to one (ranging from 0.89 to 1.00), except for the 2012–2013 transition (with the technology change index equal to 1.23), while with the SFA approach, parameter T was negative (− 0.0174), suggesting negative technological change.

Considering the relative level of efficiency among companies (that is, the efficiency of a company compared to the most efficient ones), the two approaches suggest a slightly different message: according to DEA, on average, no companies outperformed the others in improving their efficiency, hence the gap between efficient and less efficient companies did not widen, increasingly confirming the suggestion that the sector is quite homogeneous in terms of companies’ efficiency. On the other hand, with the SFA approach, the time varying efficiency model suggested that the efficiency gap slightly opened up during time, hence the distance between efficient and less efficient companies increased.

## Conclusions

Technological innovations such as artificial intelligence (McKinsey, 2018a), blockchain (BCG, 2018) and big data (Corlosquet-Habart & Janssen, 2018) are creating new opportunities in the insurance sector, with the promise of increasing efficiency (Lin & Chen, 2020). Despite these suggestions, literature empirically assessing whether insurance companies over the past few years were able to leverage new technologies to improve efficiency is scarce.

Focusing on the US public P&C insurance sector and relying on both a nonparametric (two stage-DEA) and a parametric (SFA) approach to find evidence of higher efficiency supported by technological improvements, it emerges that on average insurance companies were not able to leverage technological innovations to improve their efficiency. These results suggest that, despite relevant opportunities and promises claimed by new technologies in the insurance sector, it is relevant to understand how to practically rely on these innovations in order to improve processes and consequently gain efficiency. Often, to reduce costs insurance companies have instead put in place cost-cutting strategies (McKinsey, 2019). Large and complex firms indeed usually take longer to fully exploit new technologies in their value chain and upskill workforce to properly benefit from them, as suggested by Damioli et al. (2021) for the case of Artificial Intelligence. Further investigating the efficiency scores, in order to understand how the relative level of efficiency among companies has changed, the two approaches suggest a slightly different message. While DEA results support that on average, no companies outperformed the others in improving their efficiency by leveraging technology, indicating that the level of relative efficiency in the industry was quite stable over time, the SFA approach shows a slightly widening gap between efficient and less efficient companies. Moreover, we found a very efficient company, a kind of leader, and a homogeneous group of followers, indicating that there is vast space for improvement for less efficient firms. Nevertheless, the lacking gap closure was not due to significant improvements of the most efficient company, that if anything worsened its efficiency during time, but to an average reduction in efficiency of its followers, suggesting that neither the leader nor on average the followers properly leveraged technology in the analyzed period in order to improve their efficiency. In a competitive scenario, where new players are entering (namely Insurtech players) and competitors need to leverage technology to improve their efficiency, a situation of inertia may seriously affect the positioning of companies, both for the leader and for the followers.

Considering the results and the mentioned promises to increase efficiency by recent technological innovations (Lin & Chen, 2020), these findings suggest the need to further investigate best practices in adopting technologies to create efficiency and, in general, to bring the promised benefits in the industry. Not just academicians, but also managers and policy makers should carefully consider the effects that a non-improvement of efficiency following technological change may have on the market structure, its competition and regulations, potentially opening to further discussion on how technological innovations should be grounded and effectively adopted or facilitated. For policy makers, this work aims at providing the basis for understanding on one hand how regulations could maximize the effect of technology on efficiency improvements, and on the other hand which measures should be put in place, depending on the view of the regulator, to either reduce the efficiency gap between companies or to consolidate the industry fostering only a few efficient players. Non-efficient insurance companies are more likely to default (Ilyas & Rajasekaran, 2019), as well as companies not leveraging technological innovations (Christensen, 2013). With a similar approach, future research should investigate on how new regulatory frameworks, business models and the changing environment are affecting efficiency for insurance companies.

## Data Availability

Not applicable.

## Footnotes

1 Even though in literature this is defined as “technical efficiency” (Cummins and Weiss, 2013), for the sake of simplicity we will refer to it as “efficiency” in this paper.

2 FactSet Fundamentals is the financial database of FactSet, a market operator with more than 40 years of activity and nearly 10.000 employees globally. Several contributions in literature relied on the FactSet database (Balboa et al., 2008; Baixauli-Soler et al., 2020; Comiran et al., 2018).

3 Additional analyses omitting the very efficient company confirm positive and significant input coefficients. Additionally, companies in this subsample slightly worsen their technology in terms of effects on efficiency during the analyzed period (i.e. coefficient of T − 0.0675, p value < 0.05). The companies’ average relative efficiency is more than twice higher than before in most of the cases (due to the absence of the very efficient company and to the consequent recalibration of scores), and BC92 suggests a fairly stable gap. Results are similar even when re-running the two-stage DEA.
